# Barriers and Facilitators Perceived by Primary Healthcare Professionals About Physical Activity Prescription, a Meta‐Ethnography

**DOI:** 10.1111/phn.70127

**Published:** 2026-05-03

**Authors:** Jesus Batuecas‐Caletrio, Beatriz Rodriguez‐Martin

**Affiliations:** ^1^ Advanced Life Support Nursing, Health Service of Castilla‐La Mancha Talavera de la Reina Spain; ^2^ Faculty of Health Sciences Department of Nursing University of Castilla‐La Mancha Talavera de la Reina Spain; ^3^ Geriatric nursing Age‐ABC Research Group University of Castilla‐La Mancha Talavera de la Reina Spain

**Keywords:** barriers and facilitators, health professionals, physical activity prescription, primary health care, qualitative research

## Abstract

**Objective:**

To examine qualitative evidence on the barriers and facilitators perceived by Primary Health Care professionals in prescribing physical activity, using the Social Ecological Model as the analytical framework.

**Methods:**

A qualitative systematic review using a meta‐ethnographic approach was conducted. Searches were performed between 2013 and 2024 in Medline, Scopus, Web of Science, ProQuest, and The Cochrane Library Plus. Two independent reviewers screened studies, applied eligibility criteria, and coded findings according to the Social Ecological Model. Methodological quality was assessed using the Joanna Briggs Institute Qualitative Assessment and Review Instrument.

**Results:**

Barriers and facilitators to physical activity prescription were identified across all five domains of the Social Ecological Model. Key barriers included time constraints, insufficient training, lack of organizational and policy support, and limited referral pathways. Facilitators included professionals’ willingness, interdisciplinary collaboration, community partnerships, and the availability of structured guidelines.

**Conclusions:**

Barriers to implementing physical activity prescription remain more prominent than facilitators. Effective implementation requires a comprehensive, system‐level strategy involving healthcare organizations as well as local, regional, and national structures to support both the management and prevention of noncommunicable diseases.

**Trial Registration:**

PROSPERO under the number CRD42024613298. https://www.crd.york.ac.uk/prospero/

**Abbreviations**
GPs: General Practitioners
NCD: Noncommunicable diseases
PA: Physical activity
PHC: Primary Healthcare
PAP: Physical activity prescription
SEM: Social‐Ecological Model.
WHO: World Health Organisation

## 1 Introduction

Physical inactivity is the fourth leading risk factor for global mortality (6% of deaths), followed by high blood pressure, tobacco use, and high blood glucose (*Global Status Report on Noncommunicable Diseases* 2014, 2014), which is rising worldwide with major implications for the prevalence of noncommunicable diseases (NCDs). So, global recommendations addressing the amount of physical activity (PA) needed for the prevention of NCDs are crucial (Global Recommendations on Physical Activity for Health 2010).

An approach to the barriers and facilitators perceived by Primary Healthcare professionals (PHC) is important, as those can hinder or contribute to decision‐making regarding PA interventions (Nathan et al. 2018) and enhance the desirable results to improve the assistance to users (Waltz et al. 2019). However, perception of health professionals and managers related to barriers and facilitators in implementing physical activity prescription (PAP) still remains unclear in PHC (Cane et al. 2012; Cowdell and Dyson 2019), and preexisting conditions and weight management were the main reasons for general practitioners (GP) to advise PA, rather than using it for prevention (Patel et al. 2011).

### 1.1 Background

For long, exercise has been considered a healthy lifestyle pursuit but not a central part of medicine (Orchard 2020), and the beneficial relationship between exercise and health has been seen since the Hippocrates writings in the 5th century before Christ (Hechanova et al. 2017); but according to the existing evidence, regular PA should be the first line medicine used for both the treatment and prevention of diseases and should extend to all specialties of the healthcare systems (Sallis et al. 2015). World Health Organization (WHO) latest strategies established the importance of PA promotion by PHC, with early identification, counselling, and referrals to respond to the different needs of patients (Global Action Plan on Physical Activity 2018–2030, 2018), based on two aspects: universal access helps to reach practically all social‐economic groups, and physicians are considered the most critical source of health information (Füzéki et al. 2020). In Great Britain, over 85% of the population visit their GP at least once a year, and almost 95% do so over a 3‐year period, suggesting a great opportunity to promote PA (Pavey et al. 2011). Also, many individuals who visit healthcare for various health problems have a higher probability of being physically inactive (Andersen et al. 2022). A study found it necessary to have 12 interventions of continuous interventions a year at PHC to achieve the recommended levels of PA for a sedentary adult (Orrow et al. 2012). About responsibility for PA promotion, a study of British GPs and nurses considered activity promotion as part of their role, finding lack of time and resources as the main barriers to achieving it (Wheeler et al. 2017). Exercise on prescription and group‐based exercise were more cost‐effective than individualized gym‐based programs (Garrett et al. 2011), and interventions as advice, written materials, and referrals to exercise programs (Orrow et al. 2012) showed a cost‐utility ratio comparable to many currently funded pharmaceutical therapies (Garrett et al. 2011), so PA may be used as a complement, or even a substitute, as medication (Persson et al. 2010); needing a supporting legislative framework (Charles et al. 2022).

It is widely acknowledged that sex, age, income, and psychological variables can predict PA (Hwang and Kim 2017): Lower‐class people are less likely to engage in PA than those of higher socioeconomic status (Wilson et al. 2004). Also, socioeconomic factors affected the swimming ability of school students: middle and upper economic classes were better swimmers due to more learning opportunities (Görner et al. 2020); moreover, people with lower socioeconomic statuses are less aware of their own health problems, resulting in reduced participation in PA (Rawal et al. 2020). Because of that, we employed the Social‐Ecological Model (SEM) as a framework to layer factors that can influence human behaviors (McLeroy et al. 1988), classifying influences into 5 levels: intrapersonal, interpersonal, organizational, community, and public policies, taking views in both health research and outside the health sector (Bauman et al. 2012). The SEM was used to establish the effects of individual demographic and psychological variables, as social and physical environmental variables, indicating that all social ecological variables have significant effects (Thornton et al. 2017), adding that it encompasses a broad perspective on the dynamic interactions between people and their environment (Stokols 1992); in essence, what is actually being “treated” is the local area and not the individual per se: the balance of factors that predispose the adoption of unhealthy behaviors and those predisposing the adoption of more healthy behaviors is manipulated in favor of the latter (Cochrane and Davey 2008).

In consequence, our objective was to synthesize and analyze the quality scientific evidence that investigated barriers and facilitators perceived by PHC professionals in implementing PA.

## 2 Methods

### 2.1 Protocol and Registration

A systematic review of qualitative studies, as meta‐ethnography approach was carried out following Noblit and Hare's seven‐step process: getting started (to our research question*: What are the barriers and facilitators perceived by primary care professionals for the prescription of physical activity?*), deciding what is relevant to the initial interest (to focus only on studies of our criteria), reading the studies (to identify concepts), determining how the studies are related (looking at repeated concepts), translating the studies into one another (making a grid of concepts), synthesizing translations (making relationships between the studies), and expressing the synthesis of all the studies (W.Noblit and DwightHare 1988).

Protocol with any amendments (if applicable) was registered in PROSPERO under the number CRD42024613298.

### 2.2 Eligibility and Exclusion Criteria

The eligibility criteria used were: (1) studies reporting barriers and facilitators for prescription of PA at primary health care; (2) original studies published in English or Spanish and published in a peer‐reviewed journal between January 2013 and July 2024; (3) qualitative studies; and (4) studies analyzing perceptions of primary health care professionals (nurses, GP, physiotherapists, managers, and coordinators) to prescribe PA in adults over 18 with no previous medical conditions.

The exclusion criteria used were: (1) mixed studies where qualitative data had not been analyzed separately; (2) reviews, systematic reviews, conference abstracts, recommendations, or plans on interventions; (3) studies with PA interventions not linked to primary health care; and (4) low methodological quality studies after being analyzed by the Joanna Briggs Institute Qualitative Assessment and Review instrument (Lockwood et al. 2015).

### 2.3 Information Sources and Search Strategy

We systematically searched five electronic databases, including MEDLINE (through PubMed), Scopus, Web of Science, ProQuest, and the Cochrane Library Plus, with data published from 2013 to October 2024. Descriptors were carried out in English and combined by Boolean operators (AND and OR). Search terms used were: “physical activity,” “exercise,” “primary healthcare,” “prescription,” “barriers,” “facilitators,” and “qualitative research.” Databases were searched separately by two of us. The specific search strategies used are described in Table 1.

**TABLE 1:** Search strategy.

Database	Strategy
PubMed	(barriers) AND facilitators) AND qualitative) AND prescription) AND PA) AND primary healthcare)
Scopus	barriers AND facilitators AND qualitative AND prescription AND “physical activity” AND “primary healthcare” AND “healthcare professionals” AND PUBYEAR > 2014 AND PUBYEAR < 2025 AND (LIMIT‐TO (SUBJAREA, “MEDI”) OR LIMIT‐TO (SUBJAREA, “HEAL”) OR LIMIT‐TO (SUBJAREA, “NURS”)) AND (LIMIT‐TO (DOCTYPE, “ar”)) AND (LIMIT‐TO (LANGUAGE, “English”)) AND (LIMIT‐TO (OA, “all”)
Web of Science (WOS)	barriers and facilitators (All Fields) AND PA (All Fields) AND qualitative (All Fields) AND prescription (All Fields) AND primary health care (All Fields)
ProQuest	title(barriers AND facilitators of PA) AND fulltext (prescription) AND fulltext (primary care AND primary health care) AND fulltext (qualitative)
The Cochrane Library Plus	barriers and facilitators in Title Abstract Keyword AND “physical activity” in Title Abstract Keyword AND qualitative in Title Abstract Keyword AND prescription in Title Abstract Keyword AND “primary health care” in Title Abstract Keyword—with Publication Year from 2013 to 2024, with Cochrane Library publication date Between Jan 2013 and Jan 2024, in Trials (Word variations have been searched)

### 2.4 Data Collection Process

Two researchers carried out the search and article selection independently, following established criteria in stages (reading titles and abstracts, excluding those not eligible, and later reading articles in full to finally extract information from the selected studies). Possible inconsistencies were verified throughout all the process, and, in case of disagreement, a third researcher performed the analysis.

The information extracted from the studies consisted of study authors, country of origin, year of study and collection, objective and approach, identification of respondent professionals (sample number and professional training), characteristics of the PA intervention, data collection method, barriers, and facilitators.

The file was exported to an Excel spreadsheet customized for this study being available from the authors by request. Data was collected from studies already published and no new data generated, so informed consent was not applicable.

### 2.5 Study Selection

Studies were selected by defining barriers as any fact of a professional's situation or environment that discourages or hinders the development of skills, independence, social competence, and adaptative behavior. Facilitators were considered any fact of a person's situation or environment that encourages the development of skills, independence, social competence, and adaptative behavior (Grady et al. 2018).

Duplicate titles were excluded (automatically or manually) using the Zotero software.

### 2.6 Synthesis of Results

Two researchers combined inductive and deductive approaches in the analyses, they worked independently, extracting information line by line and coding data by using Atlas‐Ti9 software. Following the reports of the original studies, new themes were developed after categorizing data inductively. Then, these themes were deductively grouped into the five levels of the SEM, allowing to establish relations in between; so, the main themes, categories, subcategories, and codes of the barriers and facilitators for PA promotion in adults could be extracted. A provisional comparison was made with the codes identified at the previous stage, using the constant comparison method to find similarities and contradictions. Data was exported to a table containing a thematic tree, categories, subcategories, codes and cites, helping to understand the relations between the different studies (shown in additional files). The authors compared and combined the original and newly identified themes and reached a consensus on the final result.

### 2.7 Quality of Evidence and Risk of Bias Assessment

We assessed then quality of evidence of the 8 studies using the Joanna Briggs Institute Qualitative Assessment and Review Instrument (Lockwood et al. 2015) shown in Table 2. Its 10 questions with 3 options to answer (“Yes,” “No,” and “Not clear”) evaluate methodology quality for qualitative studies, congruity between the research methodology and the objectives, data collection and analysis, interpretation of results and evidences of ethical approval; with conclusions clearly flown from the interpretation of data, taking into account domains of risk of bias: consistency, directness, precision, and publication bias. Any disagreements were resolved by discussion.

**TABLE 2:** Joanna Briggs Institute Qualitative Assessment and review instrument checklist (Lockwood et al. 2015).

	1. Is there congruity between the stated philosophical perspective and the research methodology?	2. Is there congruity between the research methodology and the research question or objectives?	3. Is there congruity between the research methodology and the methods used to collect data?	4. Is there congruity between the research methodology and the representation and analysis of data?	5. Is there congruity between the research methodology and the interpretation of results?	6. Is there a statement locating the researcher culturally or theoretically?	7. Is the influence of the researcher on the research, and vice‐ versa, addressed?	8. Are participants, and their voices, adequately represented?	9. Is the research ethical according to current criteria or, for recent studies, and is there evidence of ethical approval by an appropriate body?	10. Do the conclusions drawn in the research report flow from the analysis, or interpretation, of the data?
Calonge‐Pascual et al. 2023	✓	✓	✓	✓	✓	✕	—	✓	✓	✓
Gustavsson et al. 2018	✓	✓	✓	✓	✓	✕	—	✓	✓	✓
Larsson et al. 2022	✓	✓	✓	✓	✓	✕	—	✓	✓	✓
Huijg et al. 2015	✓	✓	✓	✓	✓	✕	—	✓	✓	✓
Din et al. 2015	✓	✓	✓	✓	✓	✕	—	✓	✓	✓
Cianciara et al. 2021	✓	✓	✓	✓	✓	✕	—	✓	✓	✓
Persson et al. 2013	✓	✓	✓	✓	✓	✕	— —	✓	✓	✓
Wattanapisit et al. 2013	✓	✓	✓	✓	✓	✕	—	✓	✓	✓

*Note*: ✓, Yes; ✕, No; —unclear in the study; N/A, not applicable; JBI critical appraisal.

## 3 Results

### 3.1 Study Selection

**FIGURE 1:**
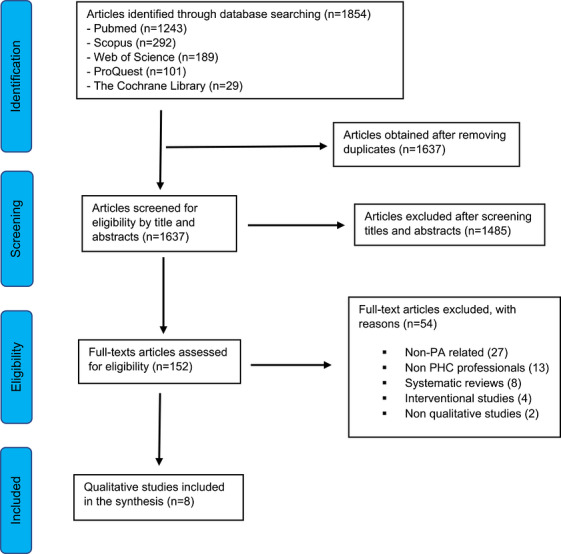
PRISMA flowchart with the studies selection process (Page et al. 2021). [Colour figure can be viewed at wileyonlinelibrary.com]

As shown in Figure 1, there were 1854 references found in the initial search, after removing 217 duplicated references, 1637 articles were reviewed on title and abstract, and of those, 1485 did not meet the inclusion criteria. Of the 152 eligible articles for which the full text was read, eight articles were finally included for data extraction.

### 3.2 Study Characteristics

The majority of studies were published in European countries (*n* = 7), followed by Asia (*n* = 1) between 2013 and 2023. Only primary health professionals were included: GPs in all of them (Calonge‐Pascual et al. 2023; Cianciara et al. 2021; Din et al. 2015; Gustavsson et al. 2018; Huijg et al. 2015; Larsson et al. 2023; Persson et al. 2013; Wattanapisit et al. 2019); nurses in 5 studies (Calonge‐Pascual et al. 2023; Cianciara et al. 2021; Din et al. 2015; Gustavsson et al. 2018; Larsson et al. 2023); physiotherapists in 2 studies (Gustavsson et al. 2018; Larsson et al. 2023); and managers and coordinators in 3 studies (Din et al. 2015; Gustavsson et al. 2018; Huijg et al. 2015). The number of participants was 168 in total, predominating more females than males (70% of females and 30% of males on average). Detailed information with country of studies, data collection techniques, and study design is described in Table 3.

**TABLE 3:** Description of the studies.

1^st^ author reference	Year	Country of study	Study design	Data collection technique	Profile professionals	Participants
(Larsson et al. 2023)	2022	Sweden	Phenomenology	Qualitative / Focus group	Nurses, GPs, physiotherapists	*N* = 24 (Females 91.6%, *n* = 22 / Males 8.4%, *n* = 2)
(Wattanapisit et al. 2019)	2019	Thailand	Phenomenology	Qualitative / Semi‐structured interview	GPs	*N* = 17 (Females 64.7%, *n* = 11 / Males 35.3% *n* = 6)
(Persson et al. 2013)	2013	Sweden	Phenomenology	Qualitative / Focus group	GPs	*N* = 15 (Females 73.3%, *n* = 11 / Males 26.7%, *n* = 4)
(Calonge‐Pascual et al. 2023)	2023	Spain	Grounded theory	Qualitative / Semi‐structured interview	GPs and nurses	*N* = 10 (Gender not indicated)
(Gustavsson et al. 2018)	2018	Sweden	Phenomenology	Qualitative / Semi‐structured interview	Managers, coordinators, GPs, nurses and physiotherapists	*N* = 18 (Gender not indicated)
(Cianciara et al. 2021)	2021	Poland	Grounded theory	Qualitative / Focus group interviews	GPs and nurses	*N* = 10 (Females 90%, *n* = 9 / Males 10%, *n* = 1)
(Din et al. 2015)	2015	UK	Grounded theory	Qualitative / Semi‐structured interview	GPs, nurses and managers	*N* = 46 (Females 56.5%, *n* = 26 / Males 43.5%, *n* = 20)
(Huijg et al. 2015)	2015	The Netherlands	Grounded theory	Qualitative / Semi‐structured interview	GPs, PHC advisors, intervention providers and managers	*N* = 28 (Gender not indicated)

Abbreviations: GPs, General Practitioners; PHC, Primary Healthcare; UK, United Kingdom.

### 3.3 Barriers and Facilitators

After analyzing data from the selected articles, two main themes emerged:

(1) Barriers and (2) facilitators for PAP perceived by PHC.

#### 3.3.1 Barriers For PAP

Five categories were identified, with several subcategories within each and their corresponding codes. Table with verbatims of barriers is shown in Table 4.

**TABLE 4:** Barriers for PAP perceived by primary health professionals.

Category	Subcategory	Code	Illustrative verbatim
Individual barriers	Attitude	Improper use of resources	“Spending money on motivating people is not justified given more serious health problems and limited resources” (Din et al. 2015).
		Uncertainty about effectiveness	“We're not convinced that PAP itself makes a difference” (Persson et al. 2013).
		Patient selection	“You've got to pick someone you think would be motivated” (Din et al. 2015).
		Gatekeeping role	“We are just gatekeepers… to prevent the system from being overwhelmed” (Din et al. 2015).
	Competence	Lack of knowledge or skills	“I am not sure whether I can choose an appropriate exercise regimen for the patient” (Wattanapisit et al. 2019).
Interpersonal barriers	Patient situation	Lack of patient motivation	“They know they should be active, but it's hard to get started” (Larsson et al. 2023).
		Patient responsibility	“Individuals should take the responsibility… not expect professionals to act as parents” (Din et al. 2015).
		Pre‐existing conditions	“We often see patients too late, at secondary rather than primary prevention” (Persson et al. 2013).
		Life routines	“Physical activity depends on life stage and responsibilities” (Calonge‐Pascual et al. 2023).
	Staff cooperation	Referrals	‘Colleagues need to realize referring patients is simple and not time‐consuming’ (Huijg et al. 2015).
		“Someone else's task”	“PA prevention should be done outside PHC settings” (Calonge‐Pascual et al. 2023).
Institutional barriers	Workload	Time constraints	“We have only 1–2 min per patient” (Wattanapisit et al. 2019).
	Organization	Lack of support	“It depends on individual commitment rather than management direction” (Gustavsson et al. 2018).
		Low priority	“There is little time for PA promotion compared to other tasks” (Din et al. 2015).
		Lack of coordination	“Support and guidance are needed to implement PA” (Gustavsson et al. 2018).
	Education	Lack of training on PA interventions	“We need education and support to implement PAP effectively” (Wattanapisit et al. 2019).
		Persistence of traditional methods	“We are trained to prioritize medical treatment, and old habits persist” (Persson et al. 2013).
Community barriers	Personal determinants	Age differences	“Younger patients may be easier to motivate than other individuals with limitations” (Din et al. 2015).
		Gender differences	“Men tend to be more physically active than women” (Calonge‐Pascual et al. 2023).
		Feelings of guilt and shame	“Some patients feel ashamed of their situation and inactivity” (Larsson et al. 2023).
	Cultural norms	Non‐prevention‐oriented culture	“Chronic disease is linked to inactivity, but prevention is not prioritised” (Din et al. 2015).
		Education received	“More educated individuals show greater awareness of their health” (Cianciara et al. 2021).
	Physical environment	Lack of facilities	“The programme is not used because facilities are not available locally” (Din et al. 2015).
Policy barriers	Healthcare system	Lack of funding	“We are asked to implement PAP but without additional resources” (Gustavsson et al. 2018).
		Role ambiguity	“There is no clear job description for this responsibility” (Gustavsson et al. 2018).
		Lack of incentives	“Remuneration does not reflect the effort required” (Cianciara et al. 2021).
		Lack of guidelines	“There is no equivalent system for PAP prescriptions” (Persson et al. 2013).
		Discontinuity of care	“Patients cannot always see the same professional” (Wattanapisit et al. 2019).
		Limited opportunities	“After leaving healthcare, patients must rely on external support systems” (Larsson et al. 2023).

##### 3.3.1.1 Individual Barriers

###### 3.3.1.1.1 Professionals’ Attitude

Some professionals stated that trying to motivate people to do something they had not done for a long time was a waste of money, which could be spent either on other more serious health problems, taking into account the current limitation of resources (Din et al. 2015). Another barrier was uncertainty among professionals as to which diseases and conditions to treat with PA and scepticism about the existing evidence for PAP, or doubts about its long‐term effect (Persson et al. 2013). Occasionally, they provided PA advice selectively, making a strictly professional diagnosis based on the results of physical checkups and by the appearance of the people, describing the active ones as satisfied, financially established, motivated, better educated, intellectual, and responsible, and the non‐active with a poor appearance as “an alcohol‐beer group” (Cianciara et al. 2021; Din et al. 2015). In other cases, professionals had feelings to serve as gatekeepers, rationing access to prevent the schemes from becoming overwhelmed, rather than actively promoting the service (Din et al. 2015).

###### 3.3.1.1.2 Competence

Healthcare professionals perceived a lack of specific knowledge and skills about supporting increased PA. In some cases there is a general lack of university training, while in others a lack of training from the current employer (Wattanapisit et al. 2019); also, there is little information on how to prescribe and dose PAP. Resulting in a lack of confidence when discussing the matter with patients (Din et al. 2015; Larsson et al. 2023).

##### 3.3.1.2 Interpersonal Barriers

###### 3.3.1.2.1 Patient Situation

Participants experienced a lack of patient motivation towards PA, based on self‐awareness, interest, and perceived barriers to PA, being surprised by the patients’ low activity levels, and a lack of a sense of individual responsibility to be fit and healthy despite professionals’ efforts to provide counselling or information (Larsson et al. 2023), seeing their role as giving advice when asked for, rather than “coercing” patients into changing their behavior (Din et al. 2015). Preexisting conditions also acted as great conditioners both physically and psychologically, with experienced obstacles to being physically active, such as pain, overweight, fear, and lack of knowledge (Persson et al. 2013); not to mention patient routines based on sedentarism and unhealthy diets (Calonge‐Pascual et al. 2023; Cianciara et al. 2021).

###### 3.3.1.2.2 Staff Cooperation

Although collaboration is widely accepted at PHC, there is a certain lack of knowledge among primary care professionals about when and how to refer patients to follow specialized assistance (Huijg et al. 2015); apart from that, there is a dichotomy in relation to if PAP has to be done by any PHC professional or if a specific PHC professional should assume the leadership in PA promotion (GPs, nurses, or physiotherapists), or even if PA preventive strategies should be done outside primary healthcare settings, as for example in sports centers, supposing a barrier we call “someone else's task” (Calonge‐Pascual et al. 2023; Persson et al. 2013).

##### 3.3.1.3 Institutional Barriers

###### 3.3.1.3.1 Workload

“Time constraint” is the factor that appears in all the articles as the main barrier for PAP; professionals perceive their workload is nearly completely dedicated to “traditional duties” such as treating patients with pathologies, prescribing and renovating medication, or asking for diagnostic tests, with little time left to inform or counsel patients about PA. Most participants, both at the management and health professional level described the PAP methods as time‐consuming, feeling they did not have sufficient time to work according to the method during normal consultations and pointing out that, so far, cost estimated for health promotion had never been taken into account in the budget preparation in the healthcare organization (Din et al. 2015; Gustavsson et al. 2018; Wattanapisit et al. 2019).

###### 3.3.1.3.2 Organization

Although central management stated that preventive strategies were a priority and a mandatory part of a healthcare delivery, PHC reported a lack of explicit managerial support for health initiatives such as PAP (Gustavsson et al. 2018). Instead, other priorities—such as smoking prevention—were perceived as receiving greater attention, leaving PA with a low level of importance (Din et al. 2015). All participants expressed the need for a designated central coordinator to provide guidance and support in developing and implementing PAP, especially during its initial phases (Gustavsson et al. 2018).

###### 3.3.1.3.3 Education

Lack of training on PA promotion, prescription, or interventions received at university was considered an important barrier that should be addressed, and regardless of the number of years in the profession, the participants agreed that medical training was geared to science and lacked teaching about non‐pharmacological methods, taking time to change a treatment strategy from prescribing drugs to replacing or supplementing this with PAP (Calonge‐Pascual et al. 2023; Gustavsson et al. 2018; Huijg et al. 2015; Larsson et al. 2023; Persson et al. 2013).

##### 3.3.1.4 Community Barriers

###### 3.3.1.4.1 Personal Determinants

Primary Health professionals referred to the relationship between PA and demographic or socioeconomic status and cognition processes, such as knowing and judging, pointing out the importance of age, gender, or place of work as determinant factors for practice of PA (Calonge‐Pascual et al. 2023; Din et al. 2015). Another element acting as a barrier in the supportive work was the patients’ feelings of guilt and shame for their unhealthy lifestyle, choices that may also have caused their current disease, supposing attitudes of resignation that make them not even try to live healthier (Calonge‐Pascual et al. 2023; Huijg et al. 2015; Larsson et al. 2023; Persson et al. 2013).

###### 3.3.1.4.2 Cultural Norms

Additionally, participants pointed to a prevailing “non‐prevention‐oriented culture” that hindered efforts to promote PA, making it difficult for patients to initiate or increase their PA levels (Din et al. 2015). They also noted that patients’ educational background played a key role in how they valued and managed their own health (Cianciara et al. 2021).

###### 3.3.1.4.3 PA Spaces

This theme highlights the shortage of appropriate physical spaces within primary healthcare settings as a major barrier to promoting and enabling PA. Participants also noted the absence of formal agreements with external community facilities for patient referral, as no structured referral system currently exists (Din et al. 2015).

##### 3.3.1.5 Policy Barriers

Although the adoption of PAP as a public health strategy requires adequate funding and strong‐system‐level commitment (World Health Organisation 2020), participants reported operating without any financial support from health authorities (Gustavsson et al. 2018). They also expressed uncertainty about their specific responsibilities due to the absence of clearly defined professional roles (Gustavsson et al. 2018). No incentives were available for PAP delivery, and participants noted that they were not given staff coverage when attending relevant training courses (Cianciara et al. 2021). In addition, the greater emphasis placed on conventional drug prescriptions over PAP, along with the lack of specific guidelines, was a recurrent source of frustration (Persson et al. 2013). Discontinuity in patient follow‐up by the same provider was viewed as a limitation to effective implementation (Wattanapisit et al. 2019). Furthermore, professionals felt unsupported by health authorities when treating patients with lower socioeconomic status, which they interpreted as a lack of equitable opportunities for these populations (Larsson et al. 2023).

#### 3.3.2 Facilitators For PAP

Five categories were identified, with several subcategories within each and their corresponding codes. Table with verbatims of facilitators is shown in Table 5.

**TABLE 5:** Facilitators for PAP perceived by primary health care professionals.

Category	Subcategory	Code	Illustrative verbatim
Individual facilitators	Attitude	Willingness to PAP	“I think it's here within primary care that we should set a good example… and work with prevention” (Larsson et al. 2023).
		Linked to personal experience	“If I am telling them something I am doing myself, it makes a difference in their acceptance.” (Din et al. 2015).
	Self‐awareness	Non‐medical approach	“For people with low mood, it's much better to give them exercise than tablets” (Din et al. 2015).
		Personal satisfaction	“To follow the patient during this change process and to see it happen… that is the reward” (Larsson et al. 2023).
		Healthy patients mean less work	“Being there for patients… so maybe they'll succeed with those small steps” (Larsson et al. 2023).
Interpersonal facilitators	Patient situation	Patient expectations	“Patients were happy that the exercise programme was tailored… it increased social interaction and reduced intimidation” (Din et al. 2015).
		Management purposes	“If you refer them to the gym they feel that you have actually done something for them… they are more likely to exercise” (Din et al. 2015).
	Staff cooperation	Team collaboration	“It's crucial to work together so patients don't get stuck between professionals” (Larsson et al. 2023).
		Team satisfaction	“Physical activity level increased, healthcare use decreased… work pressure decreased” (Huijg et al. 2015).
Institutional facilitators	Organization	Preventive purposes	“Unless patients improve diet and exercise, their health will not improve” (Wattanapisit et al. 2019).
		Support from management	“We had a manager who was a physiotherapist, so she prioritised PA” (Larsson et al. 2023).
		PA counsellor	“Delivering the intervention is not easy… assistance is important” (Huijg et al. 2015).
	Tools and education	Written prescriptions	“Having it written helps patients follow what they are supposed to do” (Larsson et al. 2023).
Community facilitators	Local networks	Information campaigns	“Information is everywhere… only willingness is lacking” (Cianciara et al. 2021).
		Access/Discounts	“Reduced cost helps people start exercising… then they continue” (Din et al. 2015).
Policy facilitators	Health authorities	Professional role	“It's added a new dimension to health care” (Din et al. 2015).
		Protocols	“PAP is a great tool to for behaviour change” (Gustavsson et al. 2018).
		Socio‐political support	“Local authorities and services were involved” (Huijg et al. 2015).
		Awareness	“Professionals learned through media and colleagues” (Huijg et al. 2015).

##### 3.3.2.1 Individual Facilitators

###### 3.3.2.1.1 Professionals’ Attitude

All participants groups (nurses, physicians, physiotherapists, and managers) expressed a strong willingness to prescribe PA, emphasizing that the responsibility to discuss PA lies on all healthcare professionals across the entire healthcare system, in terms of illness prevention, health promotion, and enhancing patient self‐esteem (Larsson et al. 2023). Additionally, clinicians who were physically active themselves felt that their personal engagement in exercise strengthened their credibility when advising patients (Din et al. 2015).

###### 3.3.2.1.2 Self‐awareness

There was full agreement among participants regarding the health benefits of increasing PA, with a strong preference for promoting sustainable lifestyle changes rather than relying solely on medication (Din et al. 2015; Gustavsson et al. 2018). Successfully providing patients with the necessary support was described as highly rewarding and motivating for their professional practice (Huijg et al. 2015; Larsson et al. 2023). Moreover, they acknowledged that healthier patients ultimately reduce the overall burden on the healthcare system, which in turn eases their own workload as healthcare professionals (Larsson et al. 2023).

##### 3.3.2.2 Interpersonal Facilitators

###### 3.3.2.2.1 Patient Situation

Patients had good expectations about PAP especially when they were taken through an individual stepped approach to improvement and increasing duration, and when being involved in classes with people in a similar situation, getting to reduce the intimidation that referred patients might feel when entering the gym, particularly for socially isolated people, which in turn gave them confidence and empowered them to modify their behavior (Din et al. 2015). Up to date, professionals have used PAP for the management of existing conditions as NCD already present, including hypertension, diabetes mellitus, and dyslipidemia, focusing it on patients with poorly controlled conditions and, in second place, with musculoskeletal disorders (Din et al. 2015; Persson et al. 2013; Wattanapisit et al. 2019).

###### 3.3.2.2.2 Staff Cooperation

Participants emphasized the value of interdisciplinary collaboration, noting that the responsibility of encouraging patients to increase their PA should be shared across multiple health professions to achieve better outcomes (Larsson et al. 2023), which caused a feeling of team satisfaction as patients improved both physically and psychologically, apart from decreasing workload (Huijg et al. 2015).

##### 3.3.2.3 Institutional Facilitators

###### 3.3.2.3.1 Organization

Within the organizational context, the primary facilitator for implementing PAP was its preventive focus. Strategies aimed at reducing the onset of various diseases, managing body weight, and improving physiological, biochemical and psychosocial health outcomes were seen as key drivers (Cianciara et al. 2021; Wattanapisit et al. 2019). Additionally, managerial support was identified as crucial for prioritizing PAP‐related activities (Larsson et al. 2023). As previously said, appointing a central PAP coordinator or educator with a leadership role to guide and motivate PHC was also viewed as essential for providing encouragement and ongoing professional support (Huijg et al. 2015).

###### 3.3.2.3.2 Tools and Education

PA interventions were supported by offering healthcare professionals practical resources—such as booklets and written guidance—to assist in delivering recommendations. Participants also emphasized the importance of having accessible community facilities outside the healthcare setting for patient referral, given the current absence of a structured referral system (Gustavsson et al. 2018; Huijg et al. 2015; Larsson et al. 2023).

##### 3.3.2.4 Community Facilitators

Participants recognized the promotion of PA through community‐based campaigns as a helpful facilitator for encouraging and sustaining PA participation (Calonge‐Pascual et al. 2023; Cianciara et al. 2021). They also highlighted the value of partnerships with local resources, such as public sports facilities, private gyms, and wellness centers, to offer discounted access for individuals enrolling (Calonge‐Pascual et al. 2023; Din et al. 2015).

##### 3.3.2.5 Policy Facilitators

Recognition of PAP by health authorities, as an official aspect of their role, served as a motivating factor for participants, encouraging them to take on this responsibility. These professionals expressed a preference toward promoting lifestyle change and regarded PAP programs as useful alternatives to prescribing (Din et al. 2015). In addition, the presence of centrally developed PAP protocols or guidelines helped ensure that professionals worked consistently toward the same goals, providing uniform information to patients, as all participants in management positions stated high confidence in PAP programs (Gustavsson et al. 2018). Participants emphasized the need for support from all relevant authorities—both public and private—to foster confidence in PAP as a feasible and achievable initiative. They explained that integrating PA interventions into national or local policies, along with adequate funding, would be highly beneficial; likewise, they stressed that support from PHC organizations and professionals is essential, as they are ultimately responsible for delivering these interventions to patients (Calonge‐Pascual et al. 2023; Huijg et al. 2015). Expanding knowledge of PAP programs in newspapers or health journals to all PHCs was identified as a key facilitator for achieving full implementation and ensuring system‐wide accessibility (Huijg et al. 2015).

This meta‐ethnographic revealed shared barriers and facilitators to the implementation of PAP across countries, while also highlighting regional differences shaped by healthcare system design, professional roles, and cultural norms. Across all regions, the most prevalent barriers were time constraints, insufficient training and competence, lack of managerial and policy support, and limited structured referral pathways to community resources (Din et al. 2015; Gustavsson et al. 2018; Huijg et al. 2015; Larsson et al. 2023; Persson et al. 2013; Wattanapisit et al. 2019). Professionals in every setting also reported challenges engaging patients with low motivation or sedentary lifestyles (Calonge‐Pascual et al. 2023; Cianciara et al. 2021). These obstacles appear largely systemic than cultural. However, regional differences shaped how PAP is implemented:

Nordic countries demonstrated a paradoxical pattern: strong professional motivation toward lifestyle‐based care and high credibility among physically active professionals, but persistent structural constraints, managerial ambiguity, fragmented continuity of care, and insufficient prioritization of PAP in daily workflows (Gustavsson et al. 2018; Larsson et al. 2023; Persson et al. 2013). These systems appear supportive on paper but lack organizational alignment for effective implementation.

In Western and Central Europe, barriers reflected greater sociocultural heterogeneity. Selective prescribing based on socioeconomic assumptions and scepticism about patient engagement were more prominent (Cianciara et al. 2021; Din et al. 2015). Professional disagreement about who should lead PAP (GPs, nurses, or physiotherapists) created a “someone else's task” dynamic that hindered implementation (Calonge‐Pascual et al. 2023). Additionally, limited staff coverage for training was a distinctive operational barrier in this region (Cianciara et al. 2021).

In Asia, findings pointed to significant resource constraints, including very high workloads, limited follow‐up continuity, and scarce organizational investment in PAP training (Wattanapisit et al. 2019). While professionals endorsed PAP for managing NCD, its implementation was hindered by structural and staffing limitations that were more acute than in European contexts.

Despite these regional variations, several facilitators were consistent across countries. Professionals expressed strong willingness to prescribe PA, recognized the effectiveness of PAP for chronic disease management, and valued interdisciplinary collaboration (Huijg et al. 2015; Larsson et al. 2023). Community partnerships and public health campaigns were widely perceived as supportive (Din et al. 2015). Importantly, centrally developed guidelines and protocols facilitated more coherent implementation, particularly in Nordic and Asian settings (Gustavsson et al. 2018; Huijg et al. 2015).

## 4 Discussion

Findings of our meta‐ethnography are consistent with other studies, professionals using established programs perceive prescription as more effective when it is linked to an existing chronic health condition than as a form of prevention (Patel et al. 2011). Effective PA promotion in healthcare settings is also seen to rely on professionals having the appropriate level of knowledge and skills to assess, counsel, and support their patients (Lion et al. 2019); determinants that can act both as barrier and facilitator as with two patient‐related determinants: “motivation” and “health status” that appear in the same study (C. Silva et al. 2023). The referral of primary care patients and residents towards local PA facilities, as a way to stimulate PA and increase health, is highlighted in some studies as very important (Bohman et al. 2015; Leenaars et al. 2016).

One study conducted a research about what had changed in the last decade about the promotion of PA, finding that PA promotion was considered by PHC professionals as part of their role; however, less than 40% thought they were effective at convincing patients to adopt a physically active lifestyle (Laberge et al. 2024); in order to overcome this, a study highlighted the importance of collaboration between medical and exercise professionals in addressing patient physical inactivity (Bray et al. 2023).

Main barriers to PAP by GPs and nurses are already described in another study as lack of time, lack of resources, and lack of success (McKenna 1998). Because of the huge costs of the physical inactivity, a study referred to PA to be assessed as the “exercise vital sign” and said it should be prescribed at the periodic health evaluation and at every opportunity (Frémont et al. 2014). Use of digital tools to implement PAP is also identified in another study as an important new technology resource (Mendes et al. 2020).

The figure of the counsellor and exercise professional as part of PAP programs appeared in a study previously identified (O'Brien et al. 2017). In case of the existence of chronic pain, a Scandinavian research study found a need for extra support when prescribed PA at PHC (Joelsson et al. 2017). “Lack of compliance/engagement” by patients and frustration of patients’ expectations as they expected drug treatment instead of exercise were also barriers perceived by health professionals (C. Silva et al. 2023).

Lack of systematic training programs for professionals in primary care was perceived as one of the main factors in not recommending PA for patients by all categories of respondents, and a reason to not recommend exercise by other health care professionals (Argeșanu et al. 2023), while suitably trained individuals involved in implementation were a facilitating factor to PAP implementation, with positive effects on staff, who then had realistic expectations about tasks and responsibilities (Calonge‐Pascual et al. 2024; Cooper et al. 2021).

Regarding the cost‐effectiveness of PAP, the same as in our research, a study suggested that advice interventions, such as exercise on prescription and some group‐based exercise programs, were more cost‐effective than individualized gym‐based or instructor‐led walking groups (Garrett et al. 2011). Also, other studies reported that all categories were open to a more collaborative approach, involving the healthcare systems and patients, with sharing of responsibility, but GPs reported that this was not in their area of responsibility, suggesting that other professionals, such as physiotherapists, were better placed for this work (Calonge‐Pascual et al. 2024; O'Regan et al. 2021).

Our main findings showing shared cross‐regional barriers to PAP (lack of time, limited training, weak policy support, and poor referral pathways) are strongly supported by recent researches, concluding that time pressure and insufficient training are the most frequent obstacles to delivering exercise prescription in primary care, being organizational support and referral systems key implementation determinants (Albert et al. 2020; C. S. Silva et al. 2023).

Patterns of regional differences match existing studies showing a recurring paradox in high‐income, prevention‐oriented systems: positive attitudes toward PA coexist with organizational misalignment (competing priorities, fragmented care, and unclear managerial priority) that prevents routine implementation, particularly described in European and Nordic focused studies (Leese et al. 2023; Nauta et al. 2022).

Resource‐constrained settings (reported in some Asia studies) commonly show greater practical barriers (heavier workloads, fewer staff, and less institutional investment in training and referral infrastructure). International scoping reviews emphasize that context‐sensitive system investments (staffing, protected training time, and referral links) are necessary for PAP scalability (Wolker Wolker Manta et al. 2022).

Facilitators described in our study (professionals’ willingness, team‐based approaches, community partnerships, and central guidelines) are also corroborated by literature, stressing that these motivators become effective only when supported by system‐level changes (funding, managerial prioritization, workflow integration). This convergence suggests that solutions must pair professional engagement with organizational and policy action (Bouma et al. 2022; Cooper et al. 2021).

Overall, our findings are broadly consistent with barriers and facilitators reported in previous studies. Even so, certain barriers identified here are less commonly described in the literature. Few studies describe patients’ feelings of guilt and shame related to engaging in exercise, and the discontinuity of care that may occur once the PAP process is initiated. Similarly, most studies do not highlight a facilitator identified by our participants: that healthier patients can reduce clinicians’ workload over time, which could serve as a potential incentive for adopting PAP in routine care.

### 4.1 Implications for Nursing

From university training onward, nurses acquire a holistic, person‐centered understanding of care that prepares them to engage with emerging health pathways, including the promotion and prescription of PA (International Council of Nurses 2021; Kemppainen et al. 2013). Their contribution to improvement of public health with PA counselling aimed at preventing and managing chronic conditions, reinforces the reliability and accountability of the nursing profession in benefiting society as a whole (Hébert et al. 2012).

Given their central place within health systems, nurses are well placed to integrate PAP into routine primary care consultations. This includes supporting patients with realistic PA goals, participating in specialized multidisciplinary PAP teams, motivating other professionals, addressing organizational and structural barriers, and advocating for stronger managerial commitment and resource allocation for PA promotion and prescription (Brotons et al. 2005; Grandes et al. 2009).

Regarding health policy, our meta‐ethnography suggest that PAP could be established as a legitimate treatment modality, as valid as medication, when implemented systematically within primary care, with nurses acting as key providers (Vuori et al. 2013). However, effective PAP delivery requires dedicated consultation time, modified scheduling, and institutional recognition of these expanded responsibilities. Such developments must be accompanied by supportive regulatory frameworks and adequate investment from health authorities (Orrow et al. 2012; World Health Organisation 2020).

## 5 Conclusion

The implementation of PAP is a multidimensional challenge that requires coordinated action from professionals, patients, and institutions. Current evidence shows that barriers still outweigh facilitators across individual, interpersonal, organizational, and policy domains. Structural constraints—such as limited time, insufficient training, lack of resources, and long‐established biomedical routines—continue to hinder the routine use of PAP, despite professionals’ generally positive attitudes and their recognition of its value in managing NCD.

Professionals across the reviewed studies emphasized that healthcare systems cannot shoulder the responsibility for promoting PA alone. Broader societal change is needed to normative PA as essential for health and to move away from the perception that pharmaceutical treatment is always required. This shift requires collective action involving schools, urban planners, and community organizations ‐for example, by integrating PA into school environments, expanding safe cycling and walking infrastructure, and offering affordable activity programs within neighborhoods.

Overall, the evidence points to the need for a comprehensive strategy that engages local, regional, and national stakeholders. PAP should evolve from a tool used primarily for managing existing conditions to a proactive mechanism for preventing the onset of NCD. Achieving this will require multidisciplinary teams trained in PAP, standardized written prescriptions, partnerships with community institutions, and—especially—adequate time and organizational support for implementation. Promotion PA must be understood as a shared responsibility across sectors.

Finally, while motivational facilitators appear relatively consistent across regions, the success of PAP ultimately depends on system‐level design. Managerial prioritization, sufficient staffing, coordinated workflows, and effective integration with community PA resources are key determinants of sustainable implementation.

### 5.1 Strengths and Limitations

The strengths of this review are the study of a framework to examine barriers and facilitators that can help health managers and professionals to identify lines of intervention and problems to implement prescription of PA but particularly addressed to healthy people and only from PHCs as it is the closest level to the community.

The main limitations are the relatively small number of studies analyzed, representing the self‐perception of a reduced sample of professionals mostly from European countries (7 out of 8 studies) and just one study from Asia, none from America nor Africa. Differences in culture, society, organization of health systems and political characteristics are to be considered; even the meaning of the terms “exercise” and “physical activity” can be different depending on the country as in (Wattanapisit et al. 2019). Most of the studies were excluded initially because of being designed to people already diagnosed of chronic illnesses.

## Author Contributions

Critical revisions for important intellectual content: Beatriz Rodriguez‐Martin. Data analysis: Jesus Batuecas‐Caletrio, Beatriz Rodriguez‐Martin. Data collection: Jesus Batuecas‐Caletrio. Manuscript writing: Jesus Batuecas‐Caletrio. Study design: Jesus Batuecas‐Caletrio. Study supervision: Beatriz Rodriguez‐Martin.

## Funding

This systematic review was not funded.

## Conflicts of Interest

The authors declare no conflicts of interest.

## Data Availability

The data that support the findings of this study are available from the corresponding author upon reasonable request.
